# Lymphocyte detection for cancer analysis using a novel fusion block based channel boosted CNN

**DOI:** 10.1038/s41598-023-40581-z

**Published:** 2023-08-28

**Authors:** Zunaira Rauf, Abdul Rehman Khan, Anabia Sohail, Hani Alquhayz, Jeonghwan Gwak, Asifullah Khan

**Affiliations:** 1 Pattern Recognition Lab, Department of Computer and Information Sciences, Pakistan Institute of Engineering and Applied Sciences, Nilore, 45650 Islamabad Pakistan; 2 PIEAS Artificial Intelligence Center (PAIC), Pakistan Institute of Engineering and Applied Sciences, Nilore, 45650 Islamabad Pakistan; 3 Department of Electrical Engineering and Computer Science, Khalifa University of Science and Technology, Abu Dhabi, UAE; 4 Department of Computer Science and Information, College of Science in Zulfi, Majmaah University, 11952 Al-Majmaah, Saudi Arabia; 5 Department of Software, Korea National University of Transportation, Chungju, 27469 Republic of Korea; 6 Center for Mathematical Sciences, Pakistan Institute of Engineering and Applied Sciences, Nilore, 45650 Islamabad Pakistan

**Keywords:** Cancer, Computer science

## Abstract

Tumor-infiltrating lymphocytes, specialized immune cells, are considered an important biomarker in cancer analysis. Automated lymphocyte detection is challenging due to its heterogeneous morphology, variable distribution, and presence of artifacts. In this work, we propose a novel Boosted Channels Fusion-based CNN “BCF-Lym-Detector” for lymphocyte detection in multiple cancer histology images. The proposed network initially selects candidate lymphocytic regions at the tissue level and then detects lymphocytes at the cellular level. The proposed “BCF-Lym-Detector” generates diverse boosted channels by utilizing the feature learning capability of different CNN architectures. In this connection, a new adaptive fusion block is developed to combine and select the most relevant lymphocyte-specific features from the generated enriched feature space. Multi-level feature learning is used to retain lymphocytic spatial information and detect lymphocytes with variable appearances. The assessment of the proposed “BCF-Lym-Detector” show substantial improvement in terms of F-score (0.93 and 0.84 on LYSTO and NuClick, respectively), which suggests that the diverse feature extraction and dynamic feature selection enhanced the feature learning capacity of the proposed network. Moreover, the proposed technique’s generalization on unseen test sets with a good recall (0.75) and F-score (0.73) shows its potential use for pathologists’ assistance.

## Introduction

Tumor-infiltrating lymphocytes (TILs) are a special type of human immune cells that invade the tumor microenvironment and tend to kill the cancer cells^1^. TILs appear as cells with a blue nucleus surrounded by a brown membrane in histopathological images with Immunohistochemistry (IHC) staining^2^. Localization and segmentation of TILs is considered essential in diagnostic pathology due to its use as a potential biomarker for cancer diagnosis, progression, and survival rate estimation in patients^3^.

**Figure 1:**
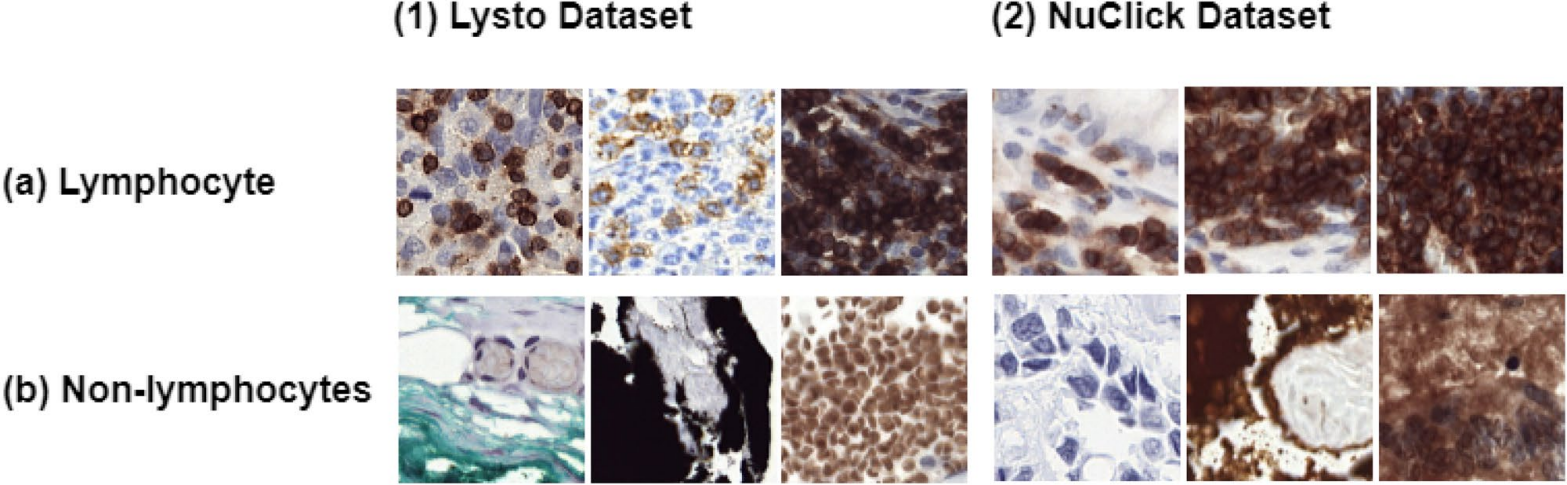
Examples from IHC stained datasets: (1) LYSTO and (2) NuClick. Panel (**a**,**b**) shows several representations of lymphocyte and non-lymphocyte containing images with highly diverse patterns, respectively.

In a standard clinical workflow, pathologists have to examine the prepared tissue slides under high magnifications and manually count TILs. This visual identification of lymphocytes is very time-consuming, tiresome, prone to human errors, and also requires pathologists to have high-level domain expertise^4,5^. Lymphocytes show high heterogeneity in their size and shape and may form clusters with unclear boundaries when many overlapping instances are present^6,7^. In addition, the presence of several staining issues, or disrupted tissue regions (known as artifacts) makes its detection even more difficult^8^ (Fig. 1). These artifacts may include tissue folds, tears, folding artifacts, or wrinkling, which can distort the tissue morphology and affect the accurate interpretation of cellular structures.

To overcome these challenges, an automated lymphocyte detection system is needed to help pathologists in their clinical workflow. Evolution in digital pathology has encouraged the development of computer-aided diagnostic approaches. Specifically, convolutional neural networks (CNNs) are widely used for various downstream tasks due to their automatic hierarchical feature extraction capability^9^, including several natural image-related tasks^10–15^. Due to the limited availability of pathologist-labeled datasets, many approaches exploit existing models pre-trained on large-sized natural image datasets and are designed for specific lab-related problems^16^.

In contrast to natural images, medical images exhibit high-level pattern diversity not only within the object of interest but also in the surrounding tissue regions^17^. These diverse patterns present high inter-class resemblance and high intra-class variations. Moreover, there are a number of overlapping and discriminant features in a single tissue region that make their detection difficult. Therefore, diagnostic systems based on a single model pre-trained on natural images may fail while performing complex tasks like lymphocyte detection.

**Figure 2:**
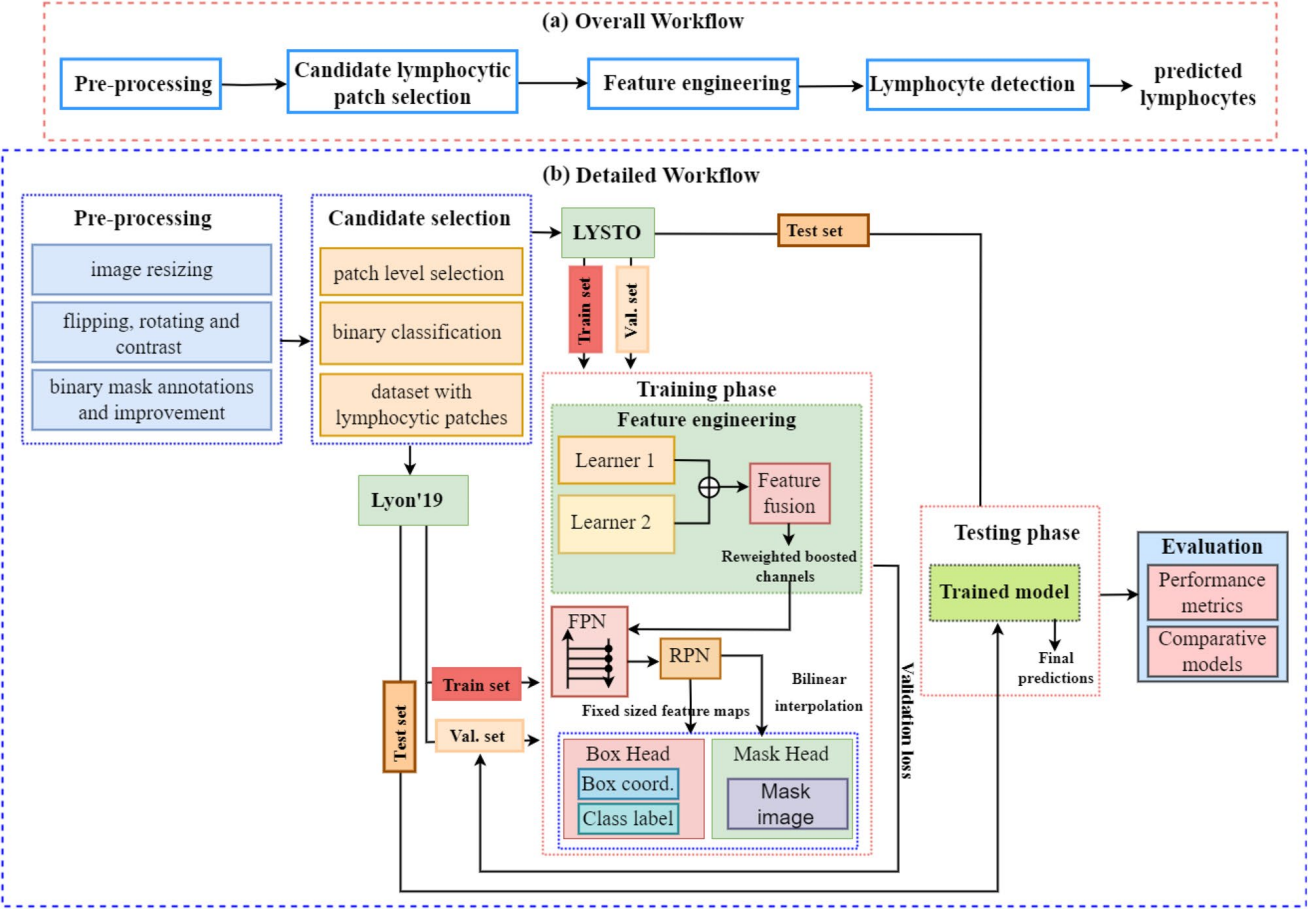
Overall (**a**), and detailed (**b**) workflow diagram of the proposed “BCF-Lym-Detector”.

Regarding the complex nature of lymphocytes, there is a need to design a specialized technique that can capture diversity in lymphocytic morphology and appearance in histopathological images. In this work, we have developed new boosted channels fusion-based CNN “BCF-Lym-Detector” for lymphocyte detection in IHC-stained histopathological images of prostate, breast, and colon cancer patients. At first, a tissue-level lymphocyte selection step is carried out to identify the tissue patches with lymphocyte candidates that are then analyzed using “BCF-Lym-Detector” to detect lymphocytes in these regions. For this purpose, we have developed a dynamic feature space by generating boosted channels from multiple CNN-based architectures to capture diverse lymphocytic patterns effectively. Multiple learners may learn overlapping features, resulting in a high-dimensional feature space. To overcome this issue, we designed a robust feature fusion technique that re-weights the learned heterogeneous features and selects the most relevant and discriminating features. The performance of the proposed “BCF-Lym-Detector” is assessed on unseen test sets containing immunohistochemistry (IHC) stained histology images. Figure 2 shows the overall workflow diagram of the developed method, where the input images were first pre-processed and then the candidate selection step was carried out. In the candidate selection step, binary classification was performed to identify lymphocytic and non-lymphocytic patches. The selected lymphocytic patches were then fed to the feature engineering module, where feature representations from two diverse learners were systematically fused using the fusion block. The selected boosted channels were then taken as input by the feature pyramid network “FPN” for learning multi-level features. Later the region proposal network “RPN” identified the probable lymphocytic regions to detect lymphocytes using the box and mask prediction head.

Our contributions are listed below:

1. A deep CNN-based detection model is proposed to detect lymphocytes in multiple cancer histology images. The proposed model combines the capacity of two independent CNN architectures to improve the feature extraction ability of the detection approach.
2. The proposed “BCF-Lym-Detector” first generates a channel-boosted feature space and then selects the most relevant and discriminating lymphocyte-specific features.
3. A novel feature fusion technique is developed to re-weight the learned diverse feature spaces by locally and globally analyzing the generated deep boosted channels.

The rests of the contents are as follows. “Related work” briefly describes the related works. Materials and the methodology of the developed “BCF-Lym-Detector” are mentioned in detail in “Materials” and “Proposed methodology”, respectively. Results and discussion are provided in “Results and discussion” and “Conclusion” finally concludes the paper.

## Related work

Detection in histopathological images is difficult due to their intricate patterns, high intra-object diversity, and inter-object similarity^17–20^. Various methods and techniques have already been proposed where multiple architectures are exploited to perform detection in histopathological images effectively^21,22^. Deep CNNs are hierarchical models that learn domain-specific image representations dynamically^10,23–25^. Therefore, these days, automated detection systems are majorly based on CNNs^26,27^. In medical image analysis, most approaches are based on existing architectures that are pre-trained on natural image datasets with little modifications^28^. Therefore, when applied to real-time medical problems, such techniques leave room for improvement.

In a study by Garcia et al., they designed a Deep CNN model to perform automated TIL detection in IHC-stained images of gastric cancer^29^. Saltz et al. did an extensive study to perform lymphocyte classification and necrosis segmentation in haematoxylin and eosin (H&E) stained images of 12 cancer types. In their study, they developed two CNNs for each task. The necrosis segmentation model was used to eliminate false positives of the lymphocyte classification model in necrotic regions^30^. Amgad et al. developed a deep learning-based method to characterize breast cancer images. They exploited VGG-based FCN to perform region and nucleus-level segmentation^31^. Rijthoven et al. performed TIL detection by exploiting YOLO architecture in IHC-stained tissue samples^6^. Swiderska-Chadaj et al. in their work, exploited transfer learning to detect lymphocytes in cancer images of multiple organs^3^. Lin et al. developed a fully connected network (FCN) based Fast ScanNet to detect lymph node metastasis in histology images of breast cancer^32^. Evangiline et al. exploited faster RCNN with different backbones to count lymphocytes in histology images^33^. Lu et al. developed a framework to perform TIL characterization in breast cancer histology images. Their framework performed three tasks: (1) automated selection of region of interest (ROI); (2) U-Net model for performing lymphocyte detection; and (3) TILs counting on WSIs^34^. Huang and Racoceanu used exclusive auto-encoders for lymphocyte detection and segmentation^35^. In a study, Zafar et al. reported a two-stage technique to first classify and then detect lymphocytes in histopathological images^36^. Neghabani et al. proposed PathoNet as a backbone architecture to U-Net for detecting lymphocytes and Ki-67 nuclear protein in IHC-stained breast cancer images^37^. Shaban et al. carried out automated stromal TILs’ counting in H&E-stained whole-slide images of head and neck cancer patients. In their work, they performed deep learning-based patch classification in the first stage, and then spatial co-occurrence statistics was calculated^38^. A dense dual-task network was developed by Zhang et al., to detect and segment TILs in breast cancer histopathological images. Two modules were designed to first detect the lymphocytes and then to separate their boundaries^8^. Fassler et al. utilized ResNet34 and VGG-16 for detection and segmentation to characterize TILs in whole-slide images of breast cancer^39^.

Recently, the idea of channel boosting (CB) and attention mechanism has shown tremendous performance in the field of medical images^40,41^. In CB, the learning capability of a network is enhanced by fusing the diverse features from multiple feature extractors. Merging multi-channel information facilitates the model in extracting domain-specific discriminating features^42^. The attention mechanism focuses on the salient regions and ignores the irrelevant areas of images. The feature maps undergo a dynamic weight adjustment process, where the features with high relevancy are given high weightage^43^. Most methods implementing the abovementioned ideas are designed for specific medical problems using respective lab datasets. Utilizing such techniques directly on a downstream computational pathology task like lymphocyte detection does not generalize well. Therefore, it is essential to design a new approach with some domain-specific modifications that effectively tackle the lymphocyte detection problem.

## Materials

### Dataset description

In the case of medical images, it is very difficult to obtain large labeled datasets due to their large sizes and hectic labeling procedure. In this regard, we used two diverse IHC-stained lymphocyte datasets for the development of our proposed technique to capture a wide range of diverse lymphocytic characteristics. Lymphocytes in IHC-stained patches are visible as blue nuclei surrounded by a brown membrane. The first dataset was from the LYSTO challenge^44^ which contained 20,000 tissue patches of 43 different patients with colon, breast, and prostate cancer. Images were taken at 40× magnification, i.e. ~ 0.25 micron/pixels with an image resolution of 299 × 299 pixels. Due to the invisibility of lymphocytes at border areas, the reference standard suggested a border area of 16 pixels (~ 4 μm) to be ignored while looking for a lymphocyte, resulting in an image size of 267 × 267 pixels. The second dataset was released by the NuClick developers^45^. The specimens were taken from Netherlands’ nine different hospitals. Samples of both datasets were taken from whole slide histology images of breast, prostate, and colon cancer patients. Some sample images from the datasets are shown in Fig. 1.

Both datasets were divided into train, validation, and test sets by following the hold-out cross-validation scheme. 60:20:20 ratios were kept while dividing each dataset into three splits. Any patient overlap was avoided in each of the train, validation, and test data splits to imitate real-time diagnostics and prevent over-fitting. Division details of each dataset are shown in Table 1.

**Table 1:** Dataset division scheme of lymphocyte datasets.

Dataset name	LYSTO	NuClick
Total images	15,455	871
Training dataset	9255	571
Validation dataset	3191	100
Testing dataset	3009	200

### Performance metrics

In this work, F-score along with precision and recall are reported to evaluate the effectiveness of the proposed and existing models. Due to the imbalanced nature of the lymphocyte detection task, F-score is considered the most reliable measure to evaluate the capability of the lymphocytic patch selection module and the developed “BCF-lym-Detector” (Eq. 1). This measure is an unbiased estimator and balances both recall and precision. The standard error (S.E) is computed at a 95% confidence interval using z-statistics. The correctly and wrongly detected lymphocytes in a patch image are known as true positives and false positives, respectively whereas; the missed lymphocytes are referred to as false negatives. Precision of the model measures the correctly positive predicted values from all positive predicted values (Eq. 2), whereas, recall measures the detection capacity of the model by measuring the correct predictions from the positive class (Eq. 3). For the evaluation of the candidate selection step where binary classification was carried out, we also reported MCC values (Eq. 4).

1 $$P= \frac{TP}{TP+FP}$$

2 $$R = \frac{TP}{TP+FN}$$

3 $$F=2 \times \frac{P\times R}{P+R}$$

4 $$MCC= \frac{(TP*TN-FP*FN)}{\sqrt{(\left(TP+FP\right)\left(TP+FN\right)\left(TN+FP\right)(TN+FN))}}$$

## Proposed methodology

**Figure 3:**
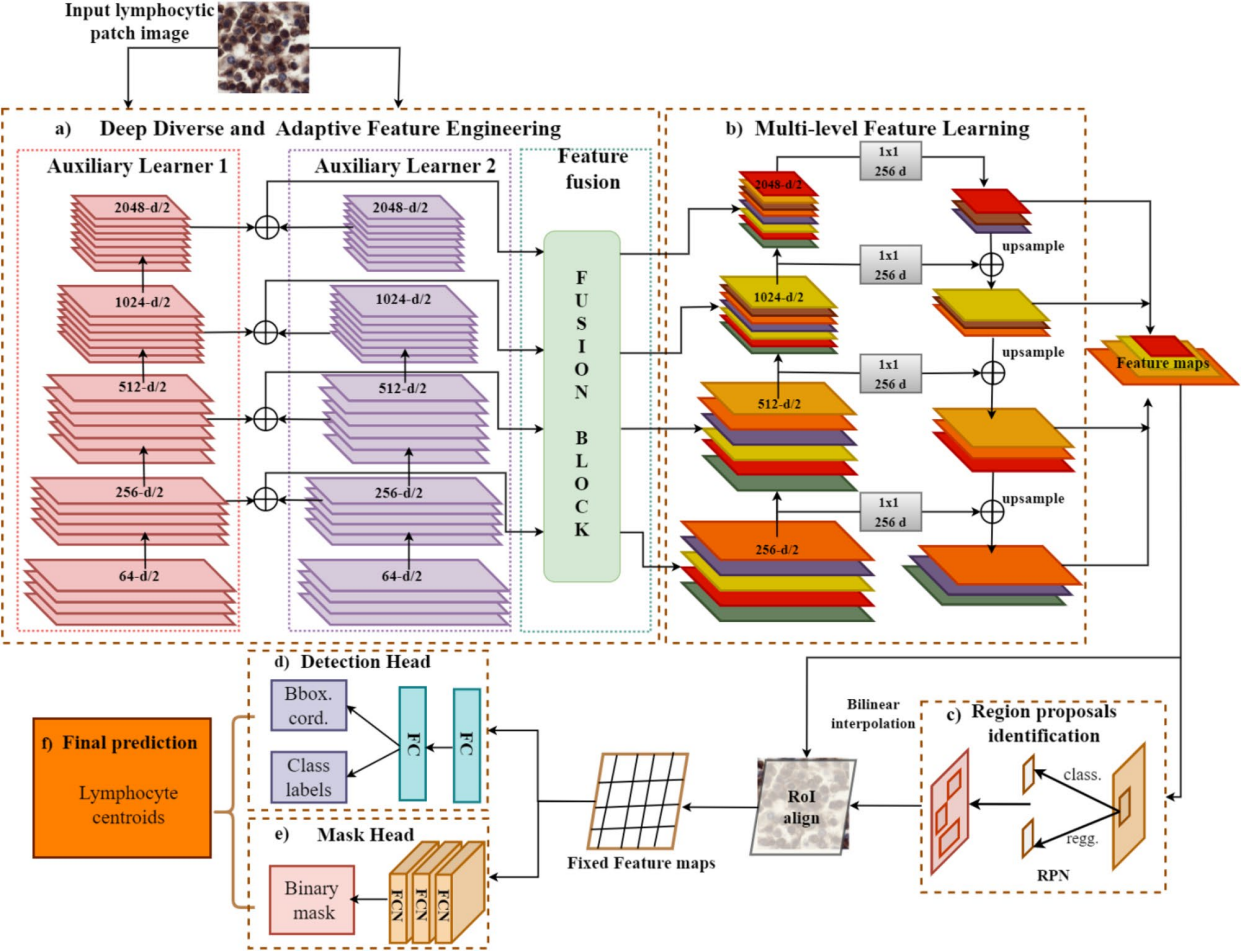
Architectural block diagram of the proposed “BCF-Lym-Detector”. After following the pre-processing and candidate selection step, the selected lymphocyte patches were fed to (**a**) the deep diverse and adaptive feature engineering stage, from where the generated features underwent (**b**) multi-level feature learning. (**c**) Region proposals were selected from the learned multi-scale and multi-level features and used by the (**d**) detection and (**e**) mask head to (**f**) detect lymphocytes.

Lymphocyte detection is a challenging task. The unavailability of large datasets, differences in data distribution, variable biological morphology, and staining artifacts make automated lymphocyte identification difficult using a single mapping function. To this end, we have proposed a boosted channels fusion block-based CNN for lymphocyte detection “BCF-Lym-Detector” in histopathological images. The stages of the proposed “BCF-Lym-Detector” are (1) data pre-processing and augmentation, (2) candidate lymphocyte selection, (3) diverse and adaptive feature engineering, (4) region proposal identification, and (5) box and mask prediction. The architectural block diagram of the developed “BCF-Lym-Detector” is shown in Fig. 3.

### Pre-processing and data augmentation

**Figure 4:**
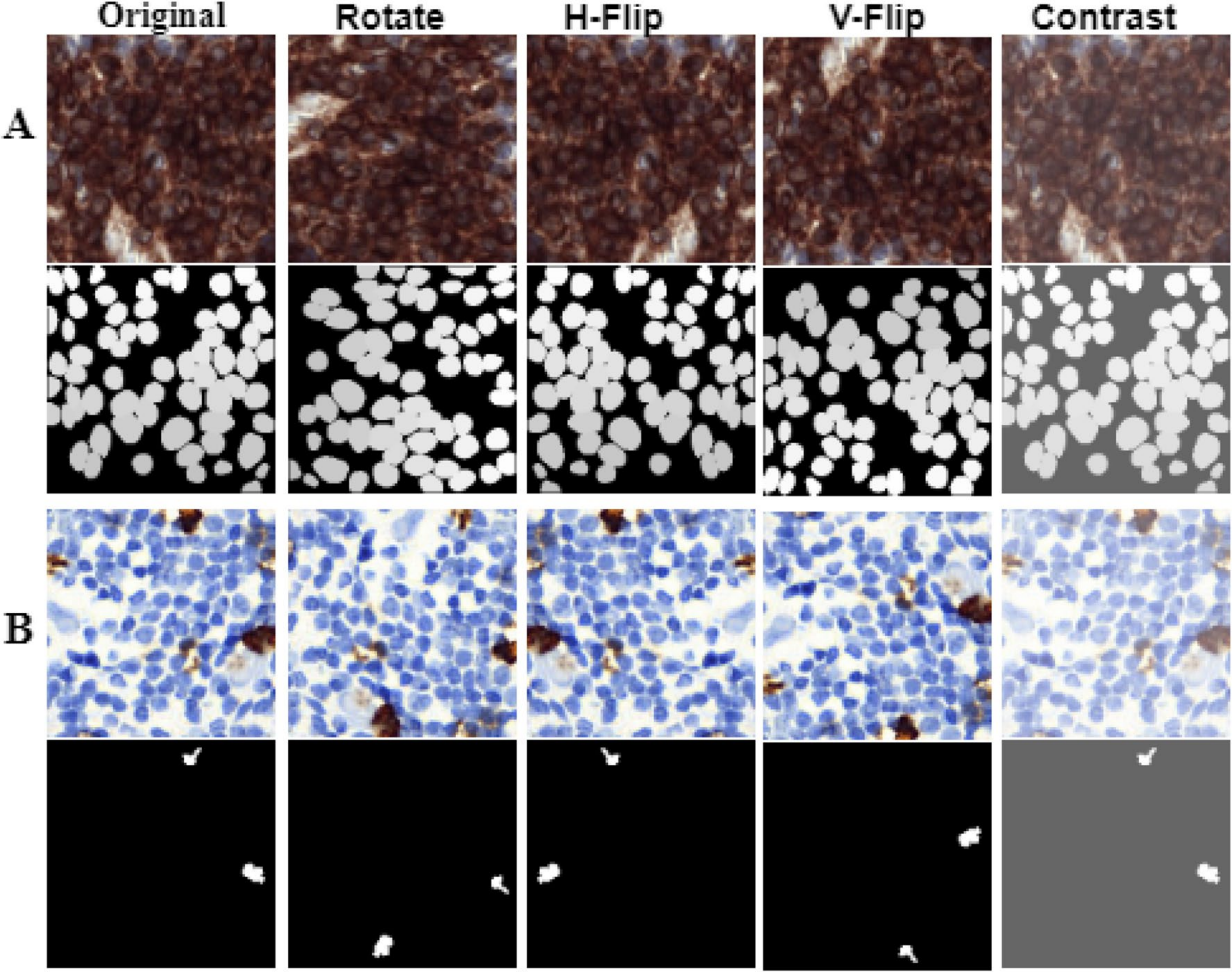
Images, augmented versions, and improved masks from (**A**) LYSTO and (**B**) NuClick datasets.

**Figure 5:**
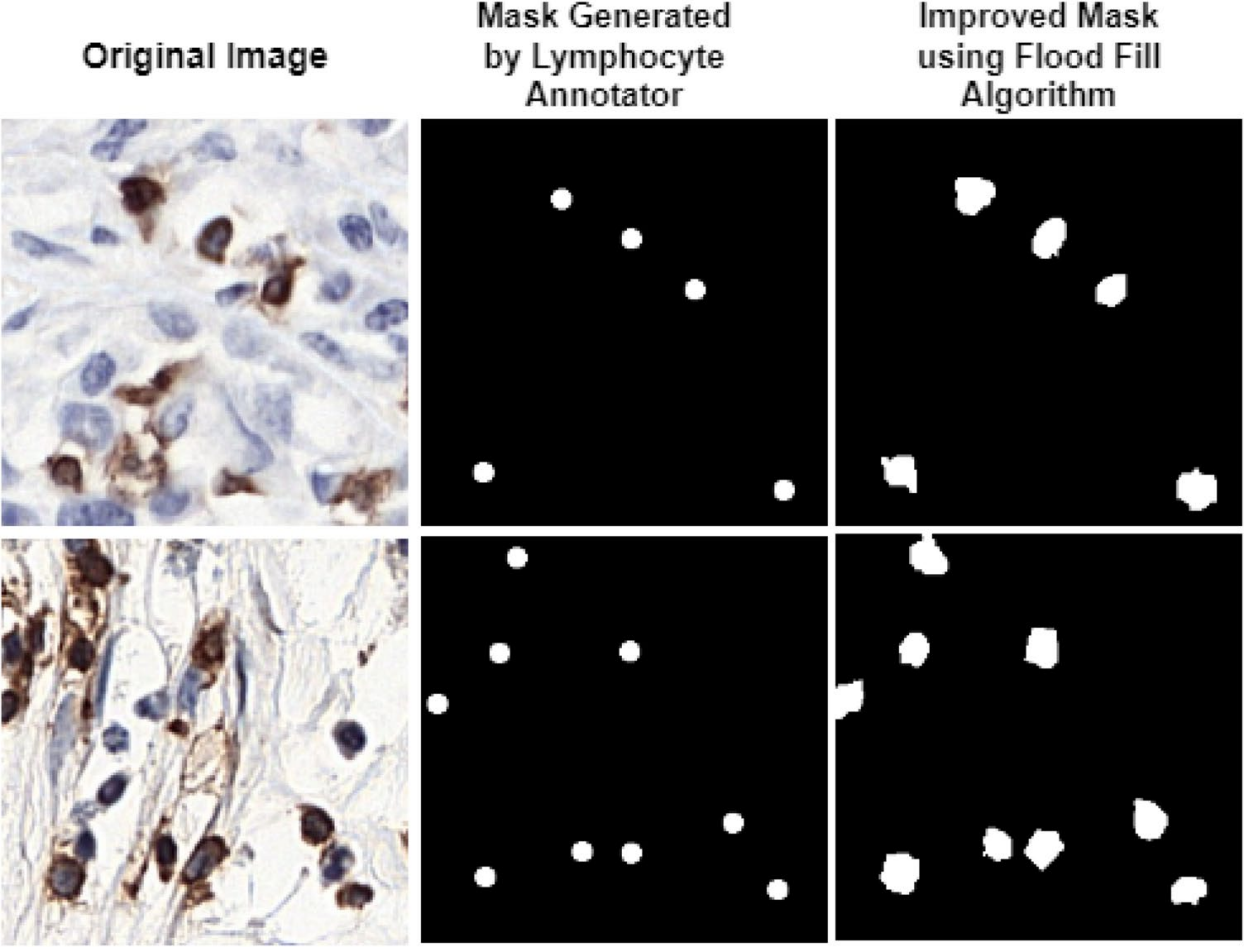
Original images (1st column), generated masks using lymphocyte annotator (2nd column), and improved masks using flood fill algorithm (3rd column).

The IHC-stained images underwent several preprocessing steps to prepare them for model training. The images were first resized to 256 × 256 pixels. Later, mean standard deviation-based image normalization was carried out before training, to have the dataset in a uniform distribution. Several augmentation techniques, including random horizontal and vertical flipping, rotation, and contrast, were applied to make the dataset more robust. Sample images of NuClick^45^ and LYSTO^44^ datasets along with their augmented versions are shown in Fig. 4A,B, respectively. Binary mask annotations were generated from the originally provided count labels for the LYSTO dataset with the help of lymphocyte annotator^46^ (Fig. 5, 2nd column). These weak masks were then further improved using flood fill region filling algorithm^47^, shown in Fig. 5, 3rd column.

### Candidate lymphocyte selection

Before feeding the dataset to the proposed detection model, an initial filtering step was performed to ignore probable image patches discriminated as artifacts. In this regard, we used ResNet50^48^ to select the candidate lymphocytic patches from non-lymphocytic patches.

### Deep diverse and adaptive feature engineering

**Figure 6:**
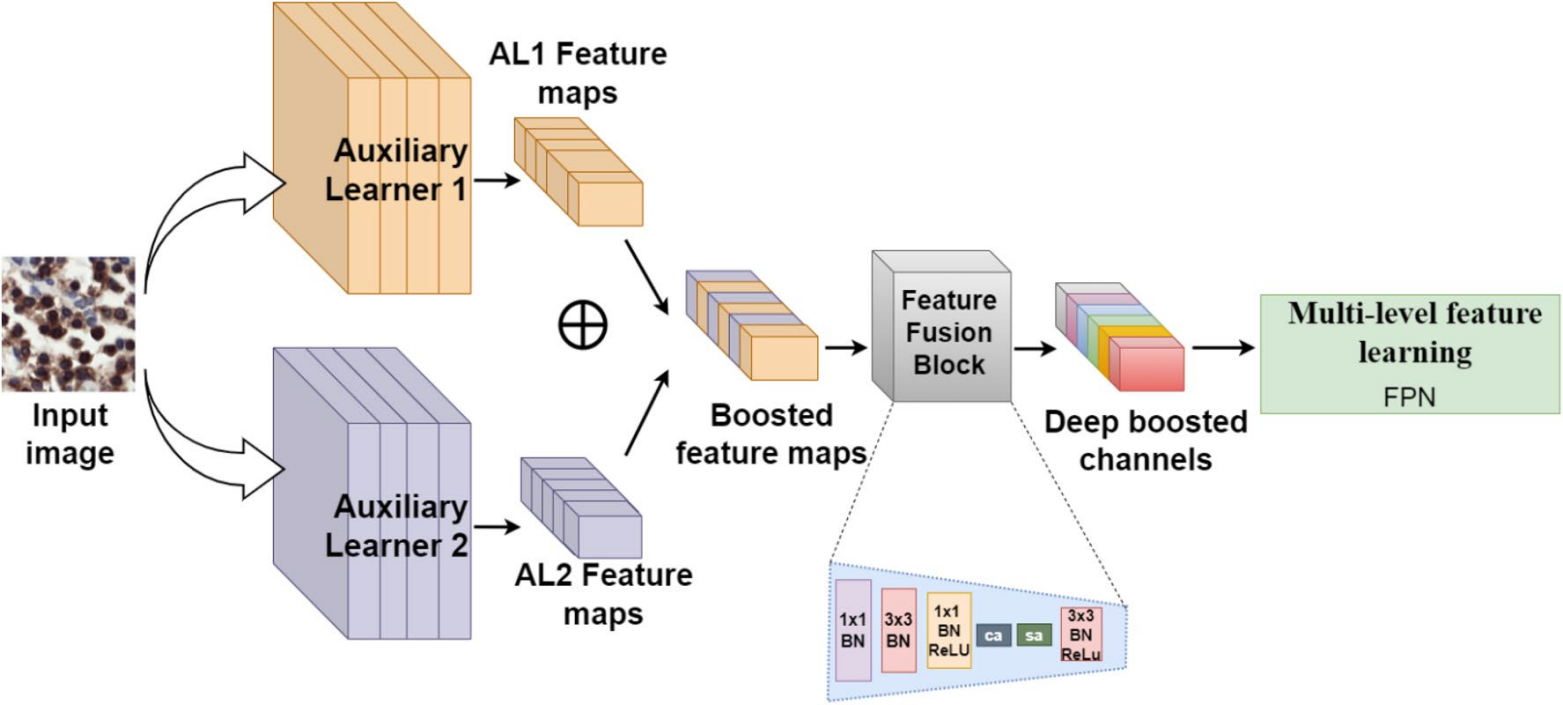
Architectural details of the proposed deep diverse and adaptive feature engineering stage. Channels from two auxiliary learners are combined using point addition to generate boosted feature maps. These boosted maps are re-weighted according to their relevance by the proposed feature fusion block to achieve deep boosted channels.

The proposed approach “BCF-Lym-Detector” aims to efficiently capture the complex lymphocytic nature by exploiting the dynamic learning abilities of different CNNs and improving the capacity of the detection model. The proposed method performs diverse and adaptive feature engineering by first generating heterogeneous boosted channels (Eq. 5) and then selecting the most domain-relevant patterns to obtain robust and refined features (Eq. 6). The block diagram of the proposed feature engineering technique is depicted in Fig. 6.

5 $${B}_{c}= {K}_{f}\left({M}_{i} ,{M}_{j}\right)$$

6 $${C}_{refined}={F}_{B}\left({B}_{C}\right)$$

where in Eq. (5), ${K}_{f}(\cdot )$ is a combiner function, that aggregates the feature spaces of auxiliary learners ${M}_{i} and {M}_{j}$ to generate a boosted channel space ${B}_{c}$. ${C}_{refined}$ in Eq. (6) is refined channel space obtained by applying the fusion block ${F}_{B}$ on the boosted channel space.

#### Diverse feature extraction

Different CNN architectures exhibit diverse feature learning capabilities. Channels from each CNN model indicate specific patterns according to their specialized blocks. Medical images display high-level pattern and textural diversity, therefore, a single CNN architecture, due to its limited learning capacity, may result in a low positive rate. Channels from multiple CNNs can be combined to achieve a boosted feature space for capturing heterogeneous patterns of lymphocytes. The idea of channel boosting involves combining the channel outputs of each learner, either through simple addition, concatenation, or using some other fusion techniques to learn different feature representations. Channel boosting can help improve the generalization and performance of deep learning models, especially when the individual models focus on different aspects or characteristics of the data. By combining the strengths and diverse perspectives of multiple models, channel boosting aims to achieve better accuracy and robustness compared to using a single model. In this regard, we have combined the feature maps of two auxiliary learners based on the idea of feature space-based knowledge transfer to analyze multiple image-specific patterns before taking the final decision by acquiring enriched feature space^49^. The features from each model are combined at every feature extraction stage using an addition operation to acquire boosted channels. The block diagram is shown in Fig. 6.

##### Auxiliary Learner 1

**Figure 7:**
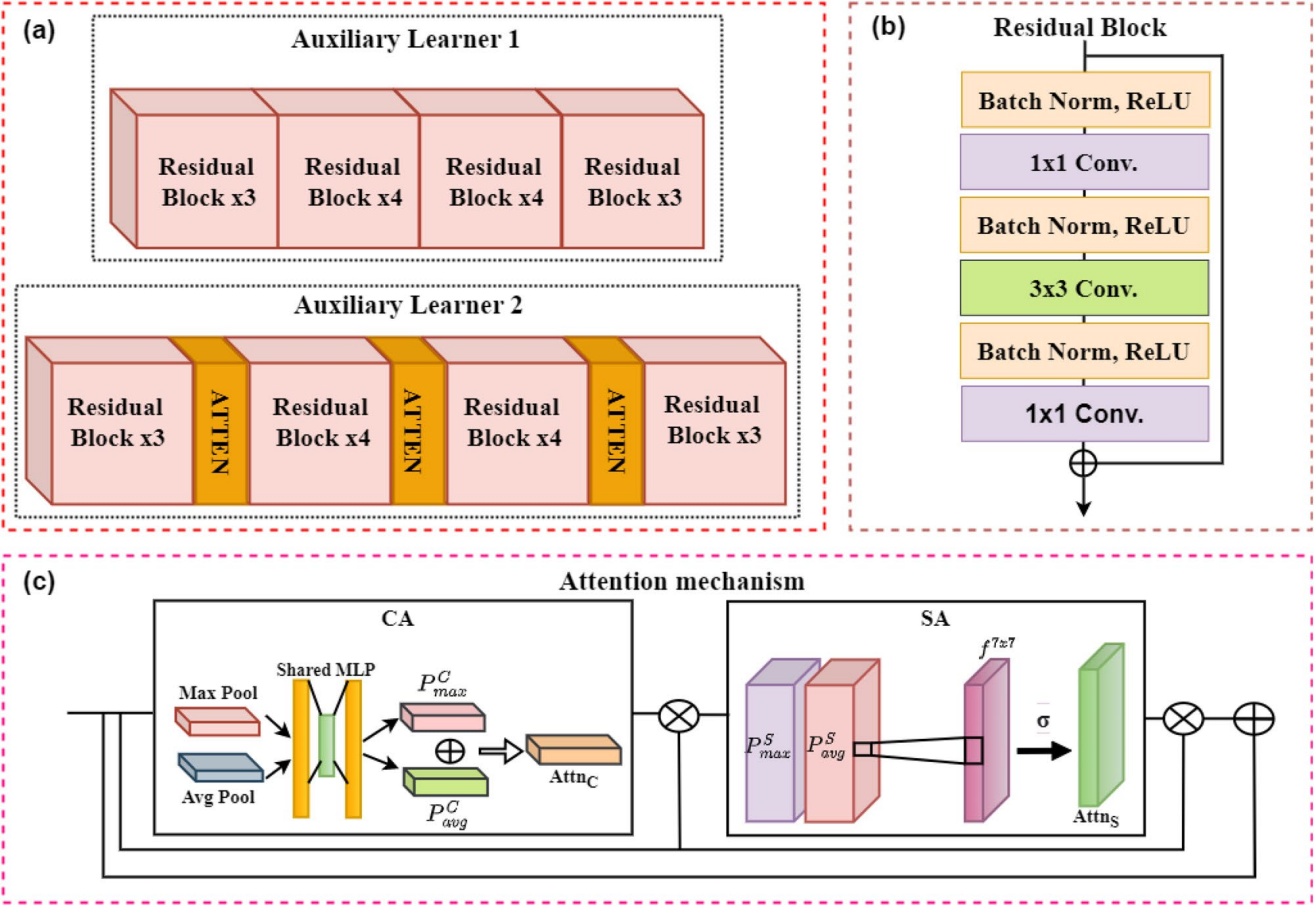
(**a**) Auxiliary learners, (**b**) ResNet architecture, and (**c**) spatial and channel attention mechanisms.

CNN models tend to mine the intricate patterns hidden in an image according to their local design. To effectively learn the dynamic mappings in lymphocytic images we aggregated two auxiliary learns to boost the feature space. The first auxiliary learner (ResNet-50) learns the non-linear function by exploiting residual blocks. The architecture of this learner is depicted in Fig. 7a. It contains multiple stages, with each stage having several residual blocks. Skip connections in these residual blocks ensure the learning of every neuron. The deep feature extraction capability of residual blocks allows the effective learning of discerning features in as complicated datasets as medical images^50^. In addition, we employed cross-domain-based transfer learning by fine-tuning the pre-trained version of ResNet-50 for feature extraction. This initialization provided a valuable starting point for our model to learn from the limited medical samples, allowing it to benefit from prior knowledge. The mathematical expression for residual connection is shown in Eq. (7). The architectural diagram of the residual block is shown in Fig. 7b:

7 $$y = f(x) + x$$

where y is the identity function obtained by adding the output of the earlier layer *x* to the output of the current layer $f(x)$.

##### Auxiliary Learner 2

This auxiliary learner is based on attention-guided transformations to ignore the irrelevant information and to emphasize the more discriminant lymphocytic features. The architecture of this learner is also based on ResNet-50 with subsequent specialized attention blocks. Like the auxiliary learner 1, this CNN-based architecture also has several feature learning stages. Its architecture is shown in Fig. 7a. The two levels of attention (1) channel attention (CA) and (2) spatial attention (SA) allowed in capturing what is important and where is the important part, respectively^51^. CA locally analyzed the feature maps across the channels to find the relevant information (Eq. 8). The average and max pooling layers compute the average and maximum value for each channel, producing a channel descriptor vector. Then the fully connected layer generates channel-wise attention weights to indicate the importance of each channel. CA allows the model to focus on more contributing channels. Whereas, SA performed depth-wise global analysis to learn the important locations in the feature maps (Eq. 9). The exploitation of these two different architectural modules boosts the feature-map representation of the proposed technique by extracting the diverse set of features. The block diagram of these two attention modules is presented in Fig. 7c:

8 $${Attn}_{c}\left({I}_{F}\right)= \sigma ({W}_{MLP}\left({P}_{avg}^{C}\left({I}_{F}\right)\right)+{W}_{MLP}({P}_{max}^{C}({I}_{F})))$$

9 $${Attn}_{s}\left({I}_{F}\right)= \sigma ({f}^{7x7}([{P}_{avg}^{S};{P}_{max}^{S}]))$$

where ${I}_{F}$ is the input feature map. ${P}_{avg}^{C}$ and ${P}_{max}^{C}$ in Eq. (8) are average and max pool operations for channel attention, respectively and ${W}_{MLP}$ is the learning function. In Eq. (9) ${P}_{avg}^{S}$ and ${P}_{max}^{S}$ are spatial average and max pool operations, and ${f}^{7x7}$ is a 7 × 7 filter used as a learning function.

#### Adaptive feature selection

The diverse and enhanced mined patterns from multiple models may also contain some redundant and overlapping information. Directly feeding these high-dimensional combined channels to a mapping function may result in poor generalization. Therefore, these multi-source abstractions are combined in a systematic way that each of them enhances the learning capability of the detection model.

##### Robust feature fusion block

**Figure 8:**
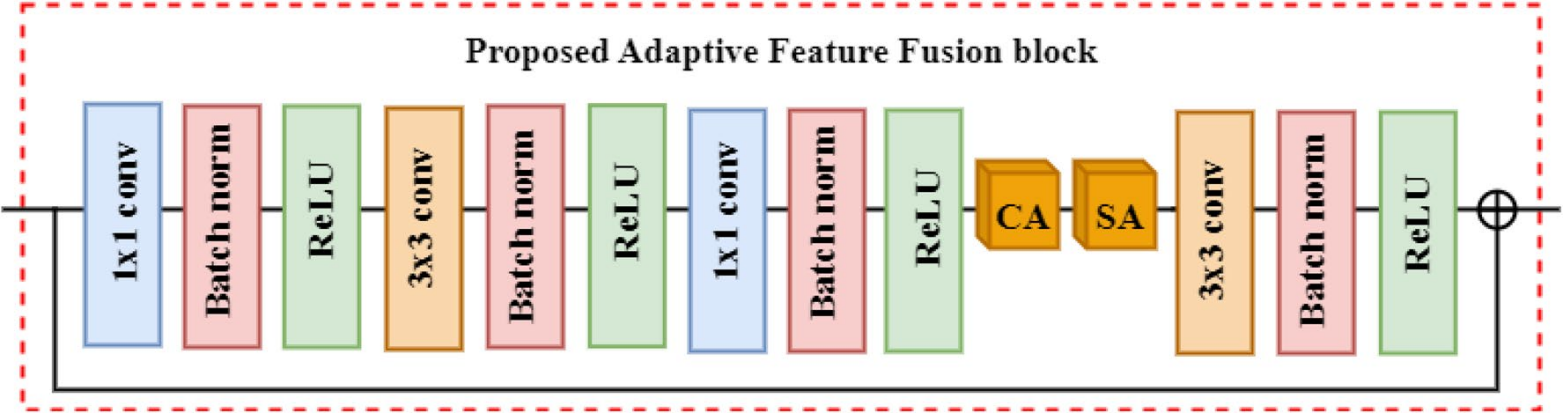
Details of proposed adaptive feature fusion block.

To further improve the semantics and reduce the dimensions of the boosted features we developed a new feature fusion block. This block performs specialized transformations to the boosted channels to capture the correlation between them and extract the most relevant information. The proposed feature fusion block takes the diverse boosted channels as input, where it applies several transformations using 3 × 3 and 1 × 1 kernels. The channel and spatial attention mechanisms in the feature fusion block allow the adaptive selection of important features by generating weight vectors for the feature maps based on high relevance. The channel attention gives positive weights to the most discriminant feature maps, emphasizing their importance, while assigning negative weights to redundant ones. On the other hand, the spatial attention mechanism assigns high weights to feature maps that contain important spatial information. These weight vectors are then multiplied with the original boosted feature maps, to effectively select only the relevant features. The feature fusion block leverages attention mechanisms to highlight the significant features while disregarding the unimportant or redundant ones. This weightage assignment ensures that only the crucial features contribute to the final representation. The proposed feature fusion block enhances the model's ability to focus on the most informative and discriminative aspects of the boosted channels, leading to improved performance in learning lymphocytic feature representations. Consequently, the proposed feature fusion block enhances the semantics and reduces the dimensions of the combined features by eliminating the influence of irrelevant components. The architectural detail of the proposed feature fusion block is shown in Fig. 8 and details of different fusion blocks are shown in Table 2.

**Table 2:** Architectural details of different fusion blocks.

Block setting	Details
Fusion block-1	2[conv3 × 3, BN, ReLU]; [ca,sa]; [conv3 × 3, BN, ReLU]
Fusion block -2	[conv1 × 1, BN, ReLU]; [conv3 × 3, BN, ReLU]; [conv1 × 1, BN, ReLU]; [ca,sa]; [conv3 × 3, BN, ReLU]
Fusion block -3	[conv1 × 1, BN, ReLU]; 2[conv3 × 3, BN, ReLU]; [ca,sa]; [conv3 × 3, BN, ReLU]

#### Multi-level feature learning

Lymphocytes exhibit variable sizes therefore feature pyramid network (FPN) is exploited in the proposed feature extraction network to incorporate the multi-scaled spatial information along with the semantic information^52^. FPN obtains a feature pyramid of different scales from each image by interpolating the feature map in a top-down manner. This enables obtaining information on different scales at different levels, thus helping in detecting objects of different sizes (Fig. 3b).

### Region proposal identification

The probable regions in the feature maps with the object of interest are identified using region proposal network (RPN) (Fig. 3c). The RPN’s classifier identifies the regions where the objects lie and its regressor gives coordinates and generates proposals for these regions. The region proposals undergo intersection over union (IoU) with the original image and only those with an overlap of 70% are selected. RPN is translational invariant, thus it incorporates robustness against translational modifications. The selected region proposals before feeding to the detection and segmentation head are resized to a fixed size by using bi-linear interpolation to preserve the spatial information.

### Box and mask prediction

The detection and segmentation heads take the fix sized feature maps to output the class labels, bounding box coordinates, and segmentation masks. The detection head contains fully connected layers followed by a softmax to predict the confidence score for each category and a regressor to perform bounding box predictions (Fig. 3d). The segmentation head has two CNNs to generate binary masks for the detected objects in the images (Fig. 3e).

### Implementation details

The proposed detector was trained for 30 epochs with a batch size of 2 and an Adam optimizer. Cross-entropy loss function and 0.0025 learning rate were used during training. The candidate selection model was also trained for 30 epochs with batch size 4, learning rate 0.001, and Adam optimizer. The validation set was used for parameters’ optimization. The proposed and existing detection models were implemented using the PyTorch framework and were trained on a system with a GTX 1070 GPU and 8 GB RAM with the same set of parameters as the proposed model.

## Results and discussion

This work selects candidate lymphocyte patches by exploiting a well-known existing CNN architecture, ResNet50. The results of this stage are shown in Table 3. The candidate lymphocyte selection module identifies the lymphocytic patches with an effective detection rate of 89%. The tissue regions discriminated as candidate regions with lymphocyte presence are assigned to the proposed “BCF-Lym-Detector” to carry out lymphocyte detection.

**Table 3:** Performance of the candidate lymphocyte selection module on LYSTO and NuClick datasets.

Model	Dataset	F ± S.E	P	R	MCC
Candidate lymphocyte selection module	LYSTO dataset	0.88 ± 0.01	0.80	0.98	0.78
	NuClick dataset	0.97 ± 0.02	0.99	0.96	0.92

The developed method “BCF-Lym-Detector” is evaluated on two different test sets based on F-score. For the comparative analysis, the proposed method was evaluated against different existing detection models. An unseen competition test set was also used to analyse the generalization ability of the proposed “BCF-Lym-Detector” method. The detailed results are presented below.

### Performance analysis of the proposed “BCF-Lym-Detector”

Performance of the proposed “BCF-Lym-Detector” is evaluated for its effectiveness in learning the lymphocytic boundaries to detect them in IHC stained images. In this regard, the diverse and adaptive feature fusion is implemented to capture lymphocyte-specific features for effective lymphocyte detection. The results of the proposed “BCF-Lym-Detector” on both datasets are shown in Table 4. The performance analysis of the developed method using different evaluation metrics (F-score: 0.93, and 0.84) suggest that the proposed channel boosting and feature fusion improves the detection capability of “BCF-Lym-Detector” significantly.

**Table 4:** Evaluation of the proposed “BCF-Lym-Detector” on LYSTO and NuClick datasets.

Dataset	F ± S.E	P	R
LYSTO dataset	0.93 ± 0.01	0.91	0.96
NuClick dataset	0.84 ± 0.02	0.86	0.82

### Qualitative results of the proposed “BCF-Lym-Detector”

**Figure 9:**
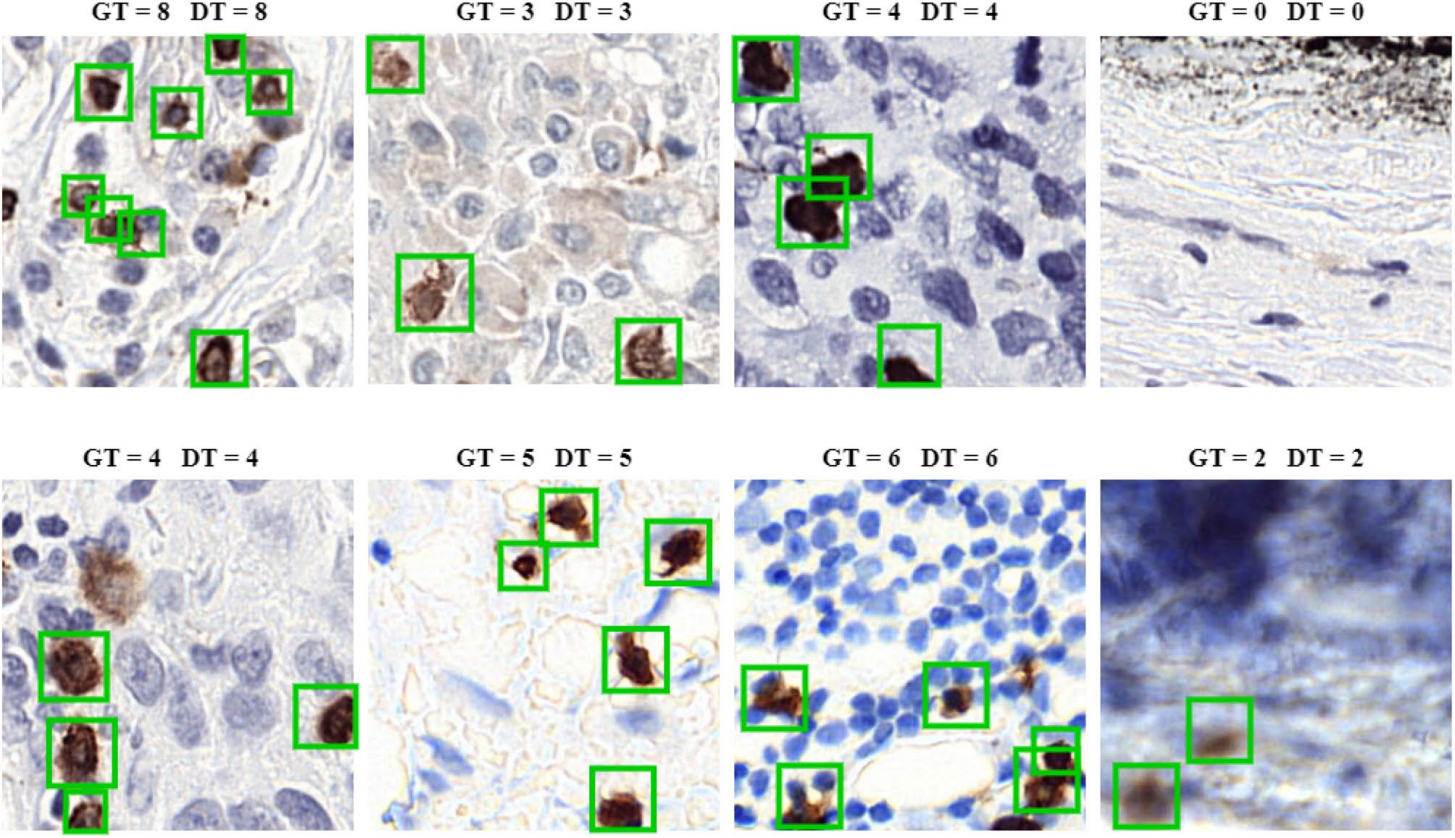
Visualization of BCF-Lym-Detector’s detection capacity on NuClick (top row) and LYSTO (bottom row), where GT means ground truth count and DT means detected count.

For a detailed assessment of the proposed “BCF-Lym-Detector” performance on the LYSTO and NuClick datasets, we carried out a qualitative analysis. Figure 9 presents a few examples to visualize the detection results of the proposed method. Results show that the proposed “BCF-Lym-Detector” has detected lymphocytes with variable shapes and sizes effectively.

### Ablation study of the proposed “BCF-Lym-Detector”

To explore the contribution of different components including auxiliary learners, fusion block, and fusion method in our proposed BCF-Lym-Detector, we performed a set of ablation experiments on the lymphocyte detection datasets. The study is conducted in a way, that different combinations of each component are tried and then its performance is compared with the proposed model. Details of these experiments are presented in the following sections.

#### Ablation experiments of auxiliary learners

First, we analysed the effectiveness of the base architectures in the proposed backbone. We implemented different combinations of ResNet50, ResNext50, ResNet101, and ResNet50 with CA and SA (Res-50-Attn) as feature extractor in the proposed method. Table 5 shows that ResNet50 and Res-50-Attn have better evaluation metric values as compared to other combinations, therefore these two architectures were selected as auxiliary learners in the proposed approach.

**Table 5:** Performance of different auxiliary learners as feature extractor in the proposed detector.

Auxiliary learners	LYSTO Dataset			NuClick Dataset		
	F	P	R	F	P	R
ResNet50 + Res-50-Attn	**0.93**	**0.91**	**0.96**	**0.84**	**0.86**	0.82
ResNet101 + Res-50-Attn	0.90	0.84	0.95	0.81	0.81	0.81
ResNet50 + ResNet101	0.89	0.84	0.94	0.82	0.81	0.82
ResNext50 + Res-50-Attn	0.84	0.89	0.80	0.82	0.80	**0.83**

Significant values are in [bold].

#### Ablation experiments of the fusion method

Second, we executed some experiments to explore the optimum method for fusing the diverse features from different architectures. Features can be fused using concatenation, like U-Net or it can be an element-wise addition, like ResNet. Therefore, to identify the effective fusion method in the lymphocyte detection task, we carried out experimentations using both fusion methods. Table 6 presents the contributions of each fusion method, and shows that feature fusion using element-wise addition has better performance than concatenation, in the proposed BCF-Lym-Detector.

**Table 6:** Performance of different fusion methods in the proposed BCF-Lym-Detector.

Fusion method	LYSTO dataset			NuClick dataset		
	F	P	R	F	P	R
Element-wise addition	**0.93**	0.91	**0.96**	**0.84**	**0.86**	0.82
Concatenation	0.87	**0.92**	0.82	0.82	0.80	**0.85**

Significant values are in [bold].

#### Ablation experiments of the proposed fusion block

Finally, a set of experiments are carried out to optimize the proposed feature fusion block. In this regard, we used three different settings of the block, with varying convolutions and attention blocks. Results show that the proposed fusion block-2 has better performance on both datasets as compared to other settings (Table 7).

**Table 7:** Results of different fusion block configurations in the proposed BCF-Lym-Detector.

Setting	LYSTO dataset			NuClick dataset		
	F	P	R	F	P	R
Fusion block-1	0.78	0.91	0.68	0.82	0.83	0.81
Fusion block-2	**0.93**	0.91	**0.96**	**0.84**	**0.86**	0.82
Fusion block-3	0.87	**0.92**	0.82	0.82	0.80	**0.84**

Significant values are in [bold].

### Performance comparison with existing detectors

There are a number of detection models that have been proposed to perform object detection in medical images. For a transparent performance evaluation of the proposed technique, we exploited several state-of-the-art models (Cascade Mask-RCNN^53^, SC-Net^54^, and YOLOx^55^) known to have good performance in the medical image domain. Due to the hungry nature of deep models, they need a large amount of training data^56^. Therefore, the already reported state-of-the-art models are fine-tuned on LYSTO and NuClick datasets by exploiting cross-domain-based transfer learning. In this regard, pre-trained ImageNet weights^57^ were used for the weight optimization of the comparative models. In addition, we also compared the proposed approach with a recent work, “DC-Lym-AF”^58^*.* Results depicted in Table 8, show that the proposed “BCF-Lym-Detector” has detected lymphocytes with a high detection rate as compared to other existing detectors.

**Table 8:** Performance comparison of the proposed “BCF-Lym-Detector” with existing detectors.

Model	LYSTO dataset			NuClick dataset		
	F ± S.E	R	P	F ± S.E	R	P
Proposed “BCF-Lym-Detector”	**0.93 ± 0.01**	0.91	**0.96**	**0.84 ± 0.05**	0.82	**0.86**
SC-Net	0.86 ± 0.01	0.81	0.90	0.83 ± 0.05	0.83	0.82
Cascade mask-RCNN	0.85 ± 0.01	0.81	0.89	0.80 ± 0.06	0.82	0.79
YOLOx	0.80 ± 0.01	0.69	0.95	0.79 ± 0.06	0.76	0.83
DC-Lym-AF	0.89	**0.93**	0.86	0.84 ± 0.05	**0.84**	0.83

Significant values are in [bold].

### Model’s robustness analysis

In practical applications, histopathological images are gathered from different labs and hospitals which leads to high variation in data distribution. The performance of machine learning algorithms is highly dependent on the underlying distribution of the training data. Therefore, an automated diagnostic system may fail when tested on real-time datasets acquired from different sources. In addition, the proposed “BCF-Lym-Detector” was evaluated on Lyon19’ competition test set to analyze its generalization ability. The challenge organizers released 441 whole slide ROI images as a test set and asked the participants to use their own training sets and to submit the test evaluation. Table 9 shows that the proposed “BCF-Lym-Detector” can detect lymphocytes with an F-score of 0.73 from an unseen test set. The proposed model was also evaluated across diverse test dataset versions, achieved by incorporating random color and rotation-based variations. The promising detection rate (0.87 and 0.83) of the proposed “BCF-Lym-Detector” shows how well it can handle variations and learn invariant features.

**Table 9:** Robustness analysis of the proposed “BCF-Lym-Detector” on variable test sets.

Dataset	F-score ± S.E	Recall	Precision
LYON-Test set	0.73 ± 0.04	0.75	0.71
LYSTO dataset with contrast and positional variations	0.87 ± 0.01	0.81	0.91
Nuclick dataset with contrast and positional variations	0.83 ± 0.05	0.85	0.81

## Conclusion

Learning strong and discriminant features is very important while detecting objects, especially in medical image analysis. TILs’ detection has an important role in cancer prognosis as they play a part in killing the tumor cells. The heterogeneous morphology of lymphocytes, the presence of artifacts, populated lymphocytes, and a high resemblance between artifacts and dense lymphocytic regions make their detection a difficult task. We have proposed a custom boosted channel fusion-based deep CNN to detect lymphocytes in IHC-stained histology images. The increase in performance of the auxiliary learners with an F-score (ranging from 0.81 to 0.84) on the NuClick test set and an F-score (ranging from 0.84 to 0.93) on the LYSTO test set shows that the proposed diverse and adaptive feature engineering technique has captured the lymphocytic diversity efficiently. The detection performance of the proposed “BCF-Lym-Detector” on an unseen test set (LYON’19) shows that it has good generalization ability. This suggests that the diverse feature space generated by the different auxiliary learners can efficiently tackle the heterogeneity and imbalance in the dataset. Moreover, transfer learning-based weight initialization of the auxiliary learners provided a valuable starting point for our model to learn from the limited medical samples, allowing it to benefit from prior knowledge. The proposed technique will provide pathologists with a second opinion and will be helpful in counting and tracking the lymphocytes. In the future, we intend to extend our work to detect other important prognostic markers for cancer diagnosis, including mitosis in variable stained histology whole slide images.

## Data Availability

All the datasets used in this research work are publically available on grand challenge website, https://grand-challenge.org/. Detailed description of the data set is provided in materials section.
